# A randomized controlled clinical trial on efficacy and safety of anakinra in patients with severe COVID‐19

**DOI:** 10.1002/iid3.563

**Published:** 2021-11-11

**Authors:** Amir Behnam Kharazmi, Omid Moradi, Mehrdad Haghighi, Mehran Kouchek, Alireza Manafi‐Rasi, Masoomeh Raoufi, ‪Simin Dokht Shoaei, Fahimeh Hadavand, Mahmood Nabavi, Mir Mohammad Miri, Sara Salarian, Seyedpouzhia Shojaei, Shayesteh Khalili, Mohammad Sistanizad, Setayesh Sadeghi, Amirhossein Karagah, Saemeh Asgari, Morteza Jaffaraghaei, Shahram Araghi

**Affiliations:** ^1^ Department of Internal Medicine, Emam Hossein Medical Center Shahid Beheshti University of Medical Sciences Tehran Iran; ^2^ Department of Clinical Pharmacy, Faculty of Pharmacy Shahid Beheshti University of Medical Sciences Tehran Iran; ^3^ Infectious Diseases and Tropical Medicine Research Center Shahid Beheshti University of Medical Sciences, Tehran, Iran Tehran Iran; ^4^ Department of Infectious Diseases, Emam Hossein Medical Center Shahid Beheshti University of Medical Sciences Tehran Iran; ^5^ Department of Pulmonary and Critical Care Medicine, Emam Hossein Medical Center Shahid Beheshti University of Medical Sciences Tehran Iran; ^6^ Department of Orthopedic Surgery, Emam Hossein Medical Center Shahid Beheshti University of Medical Sciences Tehran Iran; ^7^ Department of Radiology, Emam Hossein Medical Center Shahid Beheshti University of Medical Sciences Tehran Iran; ^8^ Prevention of Cardiovascular Disease Research Center Shahid Beheshti University of Medical Sciences Tehran Iran; ^9^ Department of Clinical Pharmacy, Faculty of Pharmacy Tehran University of Medical Sciences Tehran Iran; ^10^ Medical Biotechnology Department, Biotechnology Research Center Pasteur Institute of Iran Tehran Iran; ^11^ PersisGen Par Company Alborz Iran; ^12^ Department of Microbiology, Faculty of Advanced Science and Technology, Tehran Medical Sciences Islamic Azad University Tehran Iran

**Keywords:** acute respiratory distress syndrome, anakinra, coronavirus, COVID‐19, inflammation, interleukin‐1 inhibitor, mortality

## Abstract

**Introduction:**

Hyperinflammatory state has a role in the pathogenesis of COVID‐19. Anakinra could reduce inflammation and help to combat the condition. In this study, we aimed to assess the safety and efficacy of anakinra (PerkinRA®) in severe COVID‐19.

**Method:**

The study was an open‐label, randomized, controlled trial conducted in Imam Hossein Medical Center from May to July 2020. Patients with a confirmed diagnosis of COVID‐19 were included in this study. We administered anakinra 100 mg daily intravenously. All patients received COVID‐19 pharmacotherapy based on the represented national guideline. The need for invasive mechanical ventilation is considered the primary outcome.

**Results:**

Thirty patients were included in this study, and 15 of them received Anakinra. Nineteen patients were male (63.3%), and 11 were female (36.7%). The mean age of patients was 55.77 ± 15.89 years. In the intervention group, the need for invasive mechanical ventilation was significantly reduced compared to the control group (20.0% vs. 66.7%, *p* = .010). Also, these patients had a significantly lower length of hospital stay (*p* = .043). No significant higher rate of infection was recorded.

**Conclusion:**

Anakinra as an immunomodulatory agent has been associated with the reduced need for mechanical ventilation in patients admitted to intensive care units because of severe COVID‐19. The medication reduced the hospital length of stay. Furthermore, no increased risk of infection was observed. Further randomized placebo‐controlled trials with a larger sample size are needed to confirm these findings.

## Graphical abstract

Anakinra administration significantly reduces the need for mechanical ventilation. No increased risk of infection was observed because of anakinra use. Reduced hospitalization duration observed in patients who received anakinra.

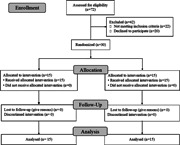

## 1 INTRODUCTION

After more than a year from the COVID‐19 pandemic, there is still a growing number of cases, and more than 3.8 million people deceased.^1^ In hospitalized patients diagnosed with COVID‐19, a higher mortality rate was observed in patients with severe disease and those with acute respiratory distress syndrome (ARDS).^2^ There is no definite treatment for COVID‐19 until the publication date, but according to the pathophysiology of the disease, many treatments have been examined, and some of them have had relatively good results.^3^ Hyperinflammatory state and cytokine storm, which is defined as excessive inflammatory mediators in patients with severe COVID‐19, play a role in the pathophysiology of COVID‐19 and ARDS.^4^ Specifically, proinflammatory interleukins (i.e., IL‐1β and IL‐6) and tumor necrosis factor‐α play a role in the cytokine storm pathway.^4^, ^5^ Pro‐IL‐1β is activated by binding severe acute respiratory syndrome coronavirus 2 (SARS‐CoV‐2) to toll‐like receptors and mediate fever, lung inflammation, and fibrosis.^6^ Anakinra is a 17‐kD recombinant nonglycosylated human IL‐1 receptor antagonist which is approved to treat rheumatoid arthritis. It could be helpful as an adjuvant treatment option in patients with severe COVID‐19 by blocking the effect of IL‐1.^7^, ^8^ There are several reports of anakinra use in sepsis, pediatric secondary hemophagocytic lymphohistiocytosis, macrophage activation syndrome, and multisystem inflammatory syndrome.^9^, ^10^, ^11^ According to the SARS‐CoV‐2 pathogenesis, this hypothesis emerged anakinra may improve the patient's situation, and in this study, we aimed to evaluate the safety and efficacy of anakinra in patients diagnosed with COVID‐19.

## 2 MATERIALS AND METHODS

This study was designed as an open‐label, randomized, controlled trial conducted at Imam Hossein Medical Center affiliated with Shahid Beheshti University of Medical Sciences, Tehran, Iran, from May 2020 to July 2020. All patients with the confirmed diagnosis of COVID‐19 based on the reverse transcriptase‐polymerase chain reaction who were admitted to the intensive care unit (ICU) were included. The study was conducted in the declaration to the Helsinki protocols, and written informed consent was given from the patients or their legal guardians before the enrollment to the trial. The study was approved by the Board of Ethics Committee (IR.SBMU.PHARMACY.REC.1399.051) and was registered in the Iranian registry for clinical trials (IRCT20120703010178N20).

The sample size of 30 patients was divided into parallel groups of intervention and control considered. Permuted block randomization with the sample size of four patients in each block which was stratified based on receiving invasive mechanical ventilation at baseline was performed. The inclusion criteria were as follows. Patients who had an age of 18 years old or more, elevated C‐reactive protein (CRP) levels, oxygen saturation less than or equal to 93% measured using a peripheral capillary pulse oximeter, fever (core temperature of 37.8°C or more), or cough or shortness of breath, and PaO_2_/FiO_2_ less than 300. Patients who had positive results for tuberculosis (i.e., positive Mendel–Mantoux or QuantiFERON test), viral hepatitis B or C, hemoglobin less than 7.5 g/dl, platelet count of fewer than 100,000 cells/µl, serum glutamic–oxaloacetic transaminase, or serum glutamic–pyruvic transaminase more than five upper limits of normal, untreated active infection, and previous administration of canakinumab or anakinra did not include to the study.

Patients in the intervention group received 100 mg anakinra (Perkinra®, PersisGen company) as an intravenous (IV) infusion once daily in addition to the standard protocol for COVID‐19 based on the sixth and seventh national COVID‐19 committee guideline, until discharge or a maximum of 14 days. In the control group, patients received medication based on the sixth and seventh national protocol released on April 29, 2020 and June 27, 2020, respectively.^12^, ^13^ The standard protocol recommended dosing and consideration of the related possible effective antiviral and immunomodulatory agents (i.e., remdesivir, lopinavir/ritonavir, interferon, favipiravir, and corticosteroid) and also, oxygen supplementation, ventilation support, fluid, and electrolyte correction, vasoactive agents and antibiotic administration, and renal replacement support if appropriate.

Individual case report forms were used for data gathering. All patients were visited daily, and data on clinical parameters and related laboratory values were recorded until discharge or expiration date. At baseline, data regarding laboratory values consisted of complete blood counts, renal and liver function tests, and inflammatory markers (i.e., CRP, erythrocyte sedimentation rate, and serum lactate dehydrogenase) were recorded. A computed tomography score was calculated based on the area of involvement to quantify the severity of pulmonary involvement. On the basis of visual inspection score of 0–5 considered for each pulmonary lobe (0 for no involvement, 1 for less than 5% of involvement, 2 for 5%–25%, 3 for 26%–49%, 4 for 50%–79%, and 5 for more than 75% of involvements).

Patients were followed to assess the primary outcome of the need for endotracheal intubation due to hypoxemia. Secondary outcomes were hospital length of stay, ICU length of stay, and seven categories ordinal scale.^14^ On Day 14, the survival for included patients was assessed.

All data were analyzed using the Statistical Package for the Social Sciences Version 20 software (IBM Company). Numerical variables are expressed as mean ± standard deviation or median (interquartile range) for parametric and nonparametric variables, respectively. Categorical data are expressed as proportions. The independent sample *t*‐test was used for parametric data and the Mann–Whitney U test was used to compare the differences in continuous variables. Differences in the categorical data were analyzed by the Chi‐square test or Fisher's exact test was performed (if more than 25% of the categories have frequencies below 5). *p* < .05 is considered as statistically significant.

The reverse power is calculated using the test for two proportions via MiniTab® statistical software (Version 18.1; 2017 Minitab, Inc).

## 3 RESULTS

**Figure 1:**
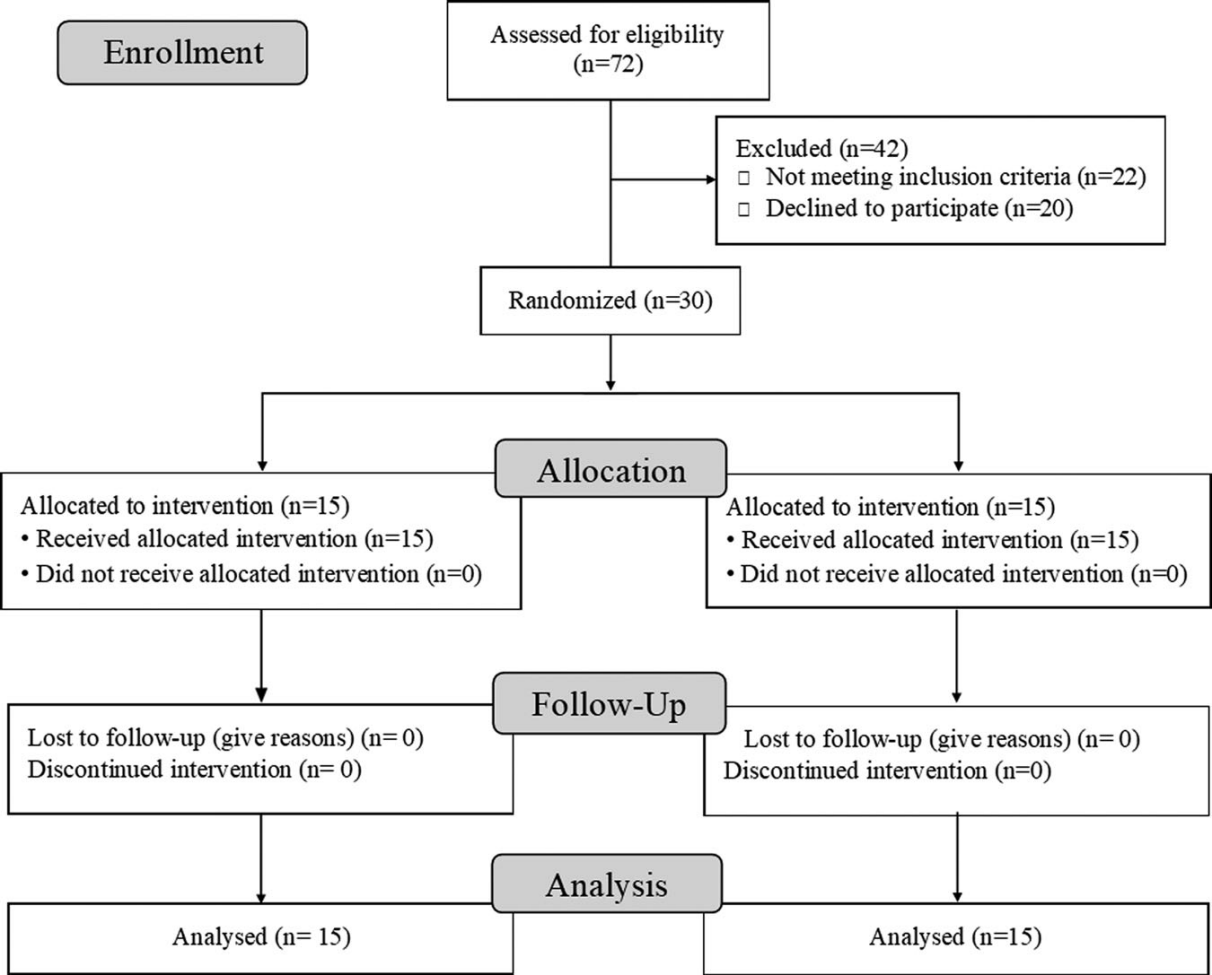
Study flow diagram

A total of 52 patients were screened to participate, and 30 patients with a mean age of 55.77 ± 15.89 years were included in the study. The study flow diagram is represented in Figure 1. The mean age was 59 years in the intervention group, and 49.25 years in the control group, 19 patients were male (63.3%), and 11 were female (36.7%) among all the participants. The minimum age of patients included in the study was 21 years, and the maximum age was 79 years.

The baseline information and clinical characteristics of all patients in anakinra and control groups are compared in Table 1. Hypertension, diabetes, and ischemic heart disease were reported as the three most prevalent medical histories of the patients. There were no significant differences in underlying conditions except for hypertension which was significantly more prevalent in the control group. The mean oxygen saturation level in the intervention group was lower but not statistically significant compared to the control group (77.33 ± 13.20 vs. 84.07 ± 6.06, *p* = .187). Patients in the intervention group had significantly higher respiratory rates (26 vs. 22, *p* = .004). No significant differences were observed in the comparison to other physical and hemodynamic properties.

**Table 1:** Baseline demographics and related clinical characteristics

Characteristics		Anakinra group (*n* = 15)	Control group (*n* = 15)	*p*
Age (years)		49.25 ± 19.12	59.00 ± 1.79	.424
Gender				
Male (%)		8	11	.309
Female (%)		7	4	
Body mass index (kg/m^2^)		28.20 ± 3.63	27.95 ± 4.93	.874
Vital signs				
Systolic blood pressure (mmHg)		129.87 ± 24.19	120.40 ± 25.82	.309
Diastolic blood pressure (mmHg)		83.87 ± 18.19	74.60 ± 11.99	.174
Pulse rate (beats/min)		96.73 ± 19.28	91.53 ± 10.97	.372
Respiratory rate (breath/min)		26 (3)	22 (5)	.004
O_2_ saturation (%)		77.33 ± 13.20	84.07 ± 6.06	.187
CT score		17.42 ± 3.73	16.15 ± 2.70	.340
Hospitalization prior enrollment (days)		4.33 ± 3.67	4.73 ± 3.75	.775
Intubated at baseline		2	2	1.000
Comorbidities				
Hypertension (%)		2 (13.3)	8 (53.3)	.020
Diabetes (%)		3 (20)	8 (53.3)	.058
Coronary artery disease (%)		3 (20)	5 (33.3)	.409
Baseline laboratory data				
WBC (cell/µl)		9200 (4100)	7900 (4830)	.420
Lymphopenia		14	10	.177
Hemoglobin (g/dl)		13.04 ± 2.18	12.01 ± 2.60	.259
INR		1.08 ± 0.10	1.10 ± 0.19	.792
PTT^15^		25.54 ± 4.93	29.80 ± 6.10	.174
Lactate dehydrogenase (U/L)		1149.46 ± 457.52	951.67 ± 408.52	.311
Ferritin (ng/ml)		780.47 ± 311.92	599.50 ± 365.39	.164
C‐reactive protein		123.69 ± 49.01	105.10 ± 51.01	.326
Erythrocyte sedimentation rate^15^		48.58 ± 23.38	68.00 ± 24.50	.065
Serum creatinine (g/dL)		1.13 ± 0.25	1.40 (0.7)	.050
Aspartate aminotransferase (U/L)		50.73 ± 19.24	32.64 ± 11.55	.005
Alanine aminotransferase (U/L)		55.86 ± 35.97	29.10 ± 15.83	.016
Medication to treat COVID‐19				
Corticosteroid		11	8	.324
Interferon		14	9	.048
Lopinavir/ritonavir		7	12	.052
Remdesivir		2	4	.505
Favipiravir		9	4	.141

Abbreviations: CT, computed tomography; INR, international normalized ratio; PTT, partial thromboplastin time; WBC, white blood cells.

Regarding laboratory markers, no significant differences were observed except for the liver function test, which was significantly higher in the intervention group, but in both groups values were lower than normal upper limits.

Patients received medications (i.e., corticosteroids, interferon β‐1a, lopinavir/ritonavir, favipiravir, and remdesivir) in pharmacotherapy regimen of COVID‐19. Except for interferon which was administered more for patients who received anakinra, no statistically significant differences were observed between the two groups.

Patients in the intervention group received anakinra for a median of 5 (3–9) days. Considering the patient's outcome, only three patients need to be intubated in the intervention group because of severe hypoxemia, and in the control group, 10 patients were intubated during their hospitalization (*p* = .010). Comparing intervention to the control group, a significant reduction of 50% in length of hospital stay (9.50 ± 4.45 vs. 19.00 ± 12.04, *p* = .043) and 67% in ICU length of stay (5.43 ± 1.72 vs. 16.60 ± 9.04, *p* = .010) was observed. The length of stay of the included patients is represented in Table 2. The endpoint of the Day 14th, represented as follows. Ten patients in the intervention group and five patients in the control group were discharged (*p* = .143). Five patients of whom received anakinra, and seven patients in the control group were deceased (*p* = .456), and two patients in the control group were still hospitalized and needed invasive mechanical ventilation (*p* = .483). Baseline, Day 7th, and Day 14th ordinal scales are presented in Table 3. As the study's main safety outcome, no episodes were observed in patients who received anakinra by examining for serious side events. Also, the infection incidence was evaluated, and three people in the control group had a positive microbiologic culture (one blood culture, two sputum cultures) confirming the infection episodes, and just one person in the intervention group (sputum culture) had an infection (*p* = .283). No other adverse events have been recorded.

**Table 2:** Length of stay (days)

	Group A (anakinra)	Group B (control)	*p*
ICU	5 (IQR = 3)	16 (IQR = 19)	.010
Hospital	10 (IQR = 5)	28.00 (IQR = 15)	.043

Abbreviations: ICU, intensive care unit; IQR, interquartile range.

**Table 3:** Ordinal scale endpoints in baseline, Day 7th and Day 14th

	Anakinra group (*n* = 15)	Control group (*n* = 15)	*p*
Baseline			
1 (%)	0 (0)	0 (0)	1.000
2 (%)	2 (13.3)	3 (20.0)	1.000
3 (%)	10 (66.7)	6 (40.0)	.272
4 (%)	3 (20.0)	6 (40.0)	.427
5 (%)	0 (0)	0 (0)	1.000
6 (%)	0 (0)	0 (0)	1.000
7 (%)	0 (0)	0 (0)	1.000
Day 7th			
1 (%)	4 (26.7)	5 (33.3)	1.000
2 (%)	1 (6.7)	5 (33.3)	.169
3 (%)	1 (6.7)	0 (0)	1.000
4 (%)	4 (26.7)	4 (26.7)	1.000
5 (%)	1 (6.7)	0 (0)	1.000
6 (%)	0 (0)	0 (0)	1.000
7 (%)	4 (26.7)	1 (6.7)	.330
Day 14th			
1 (%)	5 (33.3)	7 (46.7)	.456
2 (%)	0 (0)	2 (13.3)	.483
3 (%)	0 (0)	1 (6.7)	1.000
4 (%)	0 (0)	0 (0)	1.000
5 (%)	0 (0)	0 (0)	1.000
6 (%)	0 (0)	0 (0)	1.000
7 (%)	10 (66.7)	5 (33.3)	.143

1. Death.

2. Hospitalized, on invasive mechanical ventilation or extracorporeal membrane oxygenation.

3. Hospitalized, on noninvasive ventilation or high flow oxygen.

4. Hospitalized, requiring low flow supplemental oxygen.

5. Hospitalized, not requiring supplemental oxygen—requiring ongoing medical care (COVID‐19 related or otherwise).

6. Hospitalized, not requiring supplemental oxygen—no longer required ongoing medical care.

7. Not hospitalized.

On the basis of the result of the study's primary outcome, the reverse power was calculated and resulted in 75.92%, considering the rate of need for mechanical ventilation in the intervention group and control group are 20.0% and 66.7%, respectively. The *α* is considered as .05.

## 4 DISCUSSION

In this study, considering the primary outcome, the need for endotracheal intubation was observed in only 20% of patients receiving anakinra. In comparison, it was reported for the control group 66.7%, which indicates that the use of anakinra in patients hospitalized because of severe COVID‐19 and admitted to the ICU because of progressive hypoxemia improved their respiratory conditions. The results from all clinical trials related to anakinra have not yet been published, and some are still ongoing, but so far, the results from the published one and observational studies are consistent with the current studies.

On the other hand, as an important secondary outcome, the length of stay in the hospital was significantly shorter in the intervention group than in the control group, which is a valuable finding in the pandemic situation and the shortness of hospital bed and also, in terms of reducing the treatment costs. This result is also applied for the duration of ICU length of stay. By improving the oxygenation of patients who received anakinra, the need for invasive ventilation and using noninvasive and high flow oxygen demand were lower.

According to the studies on the effectiveness of anakinra in COVID‐19, a meta‐analysis published in lancet rheumatology included 1185 patients with moderate to severe COVID‐19 were conducted to evaluate the effect of anakinra on 28‐day mortality. This study showed anakinra administration is associated with a significant reduction in mortality (odds ratio [OR] = 0.32 [95% confidence interval, CI: 0.20–0.51]). The efficacy of anakinra was more prominent among the subgroup of patients with CRP concentrations higher than 100 mg/L.^16^

In the published clinical trial on the effect of anakinra in preventing respiratory failure in COVID‐19 patients, a significant decrease in the rate of respiratory failure was observed (22.3% vs. 59.2%, *p* < .0001). Furthermore, a considerable reduction in mortality rate was observed (4.6% vs. 12.3%, *p* = .043). Anakinra was administered by the dose of 100 mg subcutaneously once daily. The study was conducted as an open‐label nonrandomized trial. Patients needing invasive or noninvasive mechanical ventilation or PaO_2_/FiO_2_ < 150 mmHg were excluded in this study. This study confirmed that anakinra prevents respiratory failure occurs in patients with the hyperinflammation state due to COVID‐19.^17^

In the largest observational study, patients admitted to the hospital ward or ICU received a high dose of anakinra 100 mg four times a day and 200 mg three times a day, respectively. Anakinra use was associated with increased survival (OR = 3.2; 95% CI: 1.47–7.17).^15^ In another retrospective study in Italy, anakinra was used in 29 patients at a dose of 5 mg/kg iv or 100 mg twice daily subcutaneously. The results showed that the use of high‐dose IL‐1 inhibitor in patients with ARDS because of COVID‐19 is associated with a higher survival rate compared to lower doses (90% vs. 56%, *p* = .009) and is effective in improving the clinical condition of patients outside the ICU, inflammatory factors, and the need for mechanical ventilation.^18^ As an important consideration to our study, we administered anakinra IV. In the ICU setting, subcutaneous administration of the medication could be associated with unreliable pharmacokinetics.^19^

Furthermore, using immunomodulator medication in the ICU and critical care setting could be dangerous because of the increased risk of infection. It is crucial to select an agent with a lower probability of infection risk. In this study, due to the selected dose, the incidence of infection did not increase by the administration of anakinra. Previously, a more severe case of infections and bacteremia has been shown when patients received higher doses of anakinra compared to 100 mg twice daily,^18^ but with the dose of 100 mg once daily, no increase in the episodes of infection was observed. In contrast, the risk of infections is more pronounced with the other immunomodulatory agents that are being considered to treat hyper inflammation of COVID‐19, such as tocilizumab or adalimumab.^20^, ^21^, ^22^

There are some limitations to our study. First, this is a pilot study with a small sample size. Second, we did not control the trial with a placebo. It is recommended to design prospective multicentric randomized placebo‐controlled trials.

## 5 CONCLUSION

According to the study results, in general, anakinra is effective in improving the respiratory condition and significantly reduces the need for invasive mechanical ventilation in patients with severe COVID‐19. And also, the reduction was observed in hospitalization duration, which makes the medication an effective immunomodulatory agent to combat cytokine storm. As an important consideration, the reliable safety of anakinra in patients with critical conditions is one of this medication's most important properties.

## CONFLICT OF INTERESTS

The authors declare that there are no conflict of interests.

## AUTHOR CONTRIBUTIONS

*Conceptualization*: Mohammad Sistanizad, Mehrdad Haghighi, Amir Behnam Kharazmi, and Mehran Kouchek. *Methodology*: Mohammad Sistanizad, Omid Moradi, Mehrdad Haghighi, Amir Behnam Kharazmi, Amirhossein Karagah, and Masoomeh Raoufi. *Statistical analysis*: Omid Moradi and Mohammad Sistanizad. *Investigation and data curation*: Mohammad Sistanizad, Omid Moradi, Amir Behnam Kharazmi, ‪Simin Dokht Shoaei, Mahmood Nabavi, Fahimeh Hadavand, Mir Mohammad Miri, Sara Salarian, Seyedpouzhia Shoajaee, Shayesteh Khalili, Alireza Manafi‐Rasi, and Masoomeh Raoufi. *Original draft writing*: Omid Moradi, Mohammad Sistanizad, Mehrdad Haghighi, Setayesh Sadeghi, and Alireza Manafi‐Rasi. *Review and editing*: Mohammad Sistanizad, Omid Moradi, and Setayesh Sadeghi. *Supervision*: Alireza Manafi‐Rasi, Mehran Kouchek, Mir Mohammad Miri, Mahmood Nabavi, Saemeh Asgari, Morteza Jaffaraghaei, and Saemeh Araghi.

## Data Availability

The data supporting the result of this study are available on request from the corresponding author.
